# Why might medical student empathy change throughout medical school? a systematic review and thematic synthesis of qualitative studies

**DOI:** 10.1186/s12909-023-04165-9

**Published:** 2023-04-24

**Authors:** Jeremy Howick, Maya Dudko, Shi Nan Feng, Ahmed Abdirashid Ahmed, Namitha Alluri, Keith Nockels, Rachel Winter, Richard Holland

**Affiliations:** 1 Medical School, Stoneygate Centre for Excellence in Empathic Healthcare, University of Leicester, University of Leicester, George Davies Centre, Lancaster Rd, Leicester, LE1 7HA UK; 2 The University of North Carolina at Chapel Hill, Chapel Hill, NC USA; 3 Dartmouth College, Hanover, NH USA; 4 University Library, University of Leicester, Leicester, LE1 7HA UK

**Keywords:** Empathy, Systematic review, Qualitative, Communication, Medical school, Education

## Abstract

**Background:**

Several studies suggest that medical student empathy declines throughout medical school. However, no studies have synthesised the evidence regarding why empathy declines.

**Objective:**

To conduct a systematic review and thematic synthesis of qualitative studies investigating why student empathy may change throughout medical school.

**Methods:**

We included any qualitative study that investigated why empathy might change during medical school. We searched the Medline, Scopus, CINAHL, ERIC, and APA PsycInfo databases for relevant studies. All databases were searched from their inception to 18 July 2022. We also searched the reference lists of the included studies and contacted experts to identify additional studies. We used the Joanna Briggs Institute tool to evaluate the risk of bias in the included studies. Overall confidence in our results was assessed using the Confidence in the Evidence from Reviews of Qualitative research (CERQual) approach. We used thematic methods to synthesise our findings.

**Results:**

Our searches yielded 2523 records, and 16 studies involving a total of 771 students were eligible for analysis. Most studies (*n* = 11) were from Europe or North America. The descriptive themes and sub-themes were identified for each study. Increased complexity in patients and their diseases, together with the ‘hidden curriculum’ (including a stressful workload, prioritisation of biomedical knowledge, and (sometimes) poor role models), led to student adaptations, such as cynicism and desensitisation. Students’ prior lives and professional experiences appeared to exacerbate the decline in empathy. However, there were bias concerns for most of the included studies.

**Discussion:**

Many of the included studies included were small, and some did not include demographic participant data. Given the likely benefits of providing empathic care for patients and practitioners, medical education interventions should focus on developing an ‘empathic hidden curriculum’ that mitigates the decline in medical student empathy.

**Trial registration:**

A protocol for this systematic review was submitted for registration with the International Prospective Register of Systematic Reviews (PROSPERO) on 28 July 2022 (registration number CRD42022347856).

**Supplementary Information:**

The online version contains supplementary material available at 10.1186/s12909-023-04165-9.

## Background

### Rationale

Empathy in healthcare can benefit patients and practitioners. For example, it can reduce patient pain and improve care satisfaction [1]. and reduce practitioner burnout [2, 3]. The General Medical Council (GMC) in the UK, which oversees medical school training, lists empathy as a core value [4].

Despite its potential benefits, the extent to which patients report that practitioners are empathic varies [5]. Furthermore, several studies have suggested that medical student empathy appears to change throughout medical school. A systematic review published in 2011 identified 11 primary studies investigating medical student empathy change throughout medical school [6]. Most studies (*n* = 10) found that medical student empathy decreased during medical school, while one reported that empathy remained stable. A more recent systematic review published in 2020 identified 30 primary studies of empathy change throughout medical school [7]. More studies reported a decrease in empathy (*n* = 14), compared with those that found an increase (*n* = 4), no change (*n* = 6), or ambiguous results, for example, an increase on one empathy scale and a decrease on another (*n* = 6). A scoping review of 20 studies identified several problems with quantifying changes in medical student empathy, especially heterogeneity in the methods used to measure empathy change [8]. Another study suggested that empathy changes throughout medical school may involve a cultural component, with US schools demonstrating declines and Far East schools demonstrating increases in empathy [9]. There is some evidence that the decline in medical student empathy may occur in the 3rd year [10].

Qualitative studies investigating *why* empathy declines throughout medical school are rare [11]. Those that have been conducted report that the reasons for empathy decline include students’ prioritizing specialised biomedical knowledge [12] and a lack of time [11, 13]. However, the qualitative literature has not yet been synthesised, despite evidence synthesis being recommended to advance the development of medical education interventions [14].

A more comprehensive understanding of why empathy appears to decline among medical students throughout medical school can inform interventions designed to prevent or reverse the decline.

### Objective

This study aims to systematically review and synthesise the qualitative evidence investigating why medical student empathy may change throughout medical school.

## Methods

This study has been reported according to the Preferred Reporting Items for Systematic Review and Meta-Analysis Protocols (PRISMA-2020) statement [15]. A protocol for the review has been published [16].

### Eligibility criteria

We included qualitative studies that explicitly investigated why or how empathy declines throughout medical school, including outcomes related to factors that mitigate or promote empathy change throughout medical school. This included qualitative studies embedded within or reported in the same publications as non-qualitative studies (such as randomised trials, surveys, or mixed methods studies). However, no non-qualitative data was considered. Relevant qualitative studies, including interviews, focus groups, and other types of qualitative studies, such as online surveys that had relevant data, were included. Included studies could involve medical students (including undergraduate and graduate entry students) from any country. There were no language restrictions in the searches.

We excluded studies that were not qualitative, did not include medical student views, or that did not explicitly investigate the reasons for empathy change.

Following previous empirical work [1, 17–19], we included studies that explicitly used the terms ‘empathy’ or ‘empathic’. We verified the included studies using a widely accepted definition of *therapeutic* empathy, which states that it involves understanding, expression of understanding, and therapeutic action [1, 17, 20]. This definition is compatible with the emerging consensus regarding the definition of empathy [21]. This approach was limited by the fact that there are multiple and overlapping definitions of empathy, as well as the existence of other concepts, such as compassion and person-centred care, that are related to empathy [22].

### Information sources

We searched the PubMed, Embase, CINAHL, Education Resources Information Center (ERIC), and APA PsycInfo databases for relevant studies. The databases were searched from their inception to 18 July 2022. We also searched the reference lists of included studies and contacted experts to identify additional studies.

### Search strategy

We developed a search strategy using medical subject headings (MeSH) and text words related to empathy in medical school. A professional information specialist (KN) created the search strategy. Supplementary File 1 details the Medline search strategy. We used SearchRefiner to optimise the search strategy [23].

### Selection process

Relevant information about study records was uploaded to Screenatron [24] and screened by two independent reviewers (MD, AA). Disputatron was used to identify discrepancies [25], which were then resolved by a senior reviewer (JH). Two review authors (from among MD, AA, NA, SF) then independently screened the full texts to determine eligibility. Discrepancies were resolved by discussion with a third author (JH). Reasons for study inclusion or exclusion were recorded.

### Data collection process

We used a pre-piloted, standardised Excel data extraction sheet. Two independent reviewers (from among MD, AA, NA, SF) extracted the study data. Discrepancies were resolved by discussion, with an arbitrator (JH) adjudicating unresolved disagreements.

### Data items

Our primary outcome was any aspect of medical students’ reported experience or reflection of empathy in medical school, including how or why empathy might decline throughout medical school.

We extracted data about each study (aim, design, qualitative approach and rationale, data collection instruments, how data was analysed, and setting), participant characteristics (age, gender, and medical school year), interviewee (profession and characteristics), details of the interviews or focus groups (theoretical basis, interview length, and compliance); and results (descriptions and direct quotes supporting the themes and sub-themes reported by the review authors).

### Risk of bias in individual studies

We used the Joanna Briggs Institute tool to assess the risk of bias in the individual qualitative included studies [26]. This tool is considered suitable for assessing the quality of qualitative research [27]. We also assessed the risk of bias at the outcome level for each main theme. Two reviewers (from among MD, AA, NA, SF) independently checked the risk of bias for each study. Discrepancies were resolved via discussion with a senior reviewer (JH).

### Data synthesis

The population (medical students) and outcomes (exploration of why empathy declines) were sufficiently similar between studies to conduct a thematic synthesis. The scoping search suggested that the data were unlikely to be highly theoretical or conceptual. Therefore, we synthesised the data using a thematic synthesis approach, as this approach is recommended by the Cochrane Qualitative and Implementation Group for the type of data we anticipated collecting [28].

Thematic synthesis involves three phases [29], which were applied to all the included studies. Phase 1 involved line-by-line coding. Two reviewers (SNF, MD) independently coded three studies to determine meaning and context before coding the remaining studies. The codes were then discussed with a senior reviewer (JH), reviewed, and further developed. One reviewer (SNF) then coded all data, and the coding was checked by a second reviewer (MD). Discrepancies in coding were resolved through discussion with a third reviewer (JH). Phase 2 involved the generation of descriptive themes. Codes were grouped into descriptive themes and organised into a table. Using the illustrative data presented in the table, themes were captured, and similarities and differences in the data were described across different individual studies [30]. Phase 3 involved the generation of interpretive or analytical themes based on the insights gained from the synthesised data and descriptive themes. We used the NVivo software to assist with the thematic synthesis [31].

### Sub-group analysis and investigation of heterogeneity

We intended to explore possible sources of heterogeneity, including medical school programmes (graduate entry or undergraduate entry), medical student characteristics (age and sex), continent, and proportion of female students. These sub-groups were based on the hypotheses that empathy changes throughout medical school may differ by geographic region [9], that healthcare practitioner empathy varies significantly depending on practitioner characteristics (particularly sex/gender) [5] and age (i.e., whether the programme is graduate or undergraduate) [32, 33]. However, the data was not rich enough to explore these differences formally.

### Confidence in cumulative evidence

We investigated the confidence in cumulative evidence using the Confidence in the Evidence from Reviews of Qualitative Research (CERQual) approach, which is recommended by Cochrane [34]. This approach assigns a rating of ‘high’, ‘moderate’, ‘low’, or ‘very low’ confidence depending on the methodological limitations of the primary studies, the relevance of the primary studies regarding the systematic review objectives, coherence of the findings, and the adequacy of data supporting the findings. Methodological limitations were addressed by assessing the risk of bias in individual studies. Coherence was assessed by evaluating whether there was a clear fit between the data and the findings of the primary studies. Adequacy was assessed by considering whether the data from the included studies were applicable to the context specified in the review question. Relevance was assessed by examining the context of the included studies to determine whether the settings were relevant to the review question. One reviewer (AA) performed the GRADE Cerqual analysis, and another (JH) checked the ratings. We presented a summary table for each finding that includes the primary contributing studies, evaluations of the above four domains, an overall confidence rating (high, moderate, low, or very low), and a brief explanation of the rating judgement.

## Results

### Study selection

**Fig. 1:**
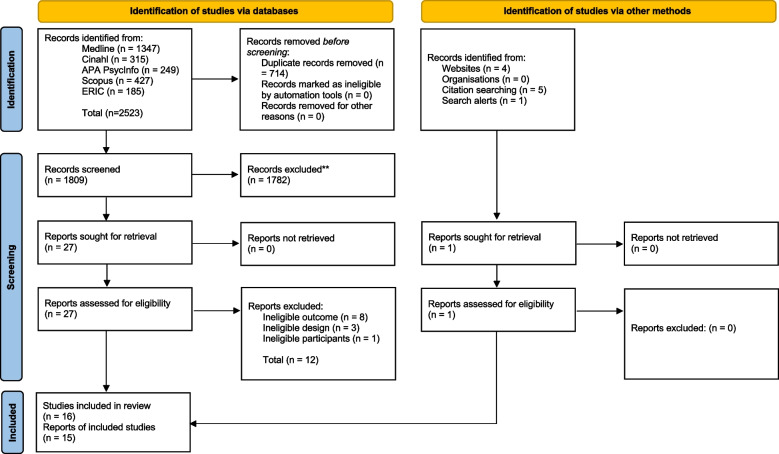
PRISMA Flow Diagram

Our search yielded 2523 publications. After duplicates were removed, we screened 1809 records. Of these, 1782 were excluded for not meeting the inclusion criteria. Ten further records were identified via our additional searches; one was eligible for full-text screening. Overall, 28 full-text records were assessed for eligibility [11–13, 35–58]. Of these, 16 were eligible for analysis (see Fig. 1) [11–13, 40, 41, 43, 44, 47–51, 53, 55, 57]. Two of the studies were published in the same paper [11].

### Excluded studies

We excluded 12 studies [35–39, 42, 45, 46, 52, 54, 56, 58] due to ineligible outcomes (did not investigate reasons for the decline in empathy; *n* = 8 [35, 37–39, 42, 46, 52, 56]), ineligible study design (non-qualitative study; *n* = 3 [45, 54, 58]), and ineligible participants (non-medical students; *n* = 1 [36]).

### Study characteristics

The 16 included studies were from Europe, including the UK (*n* = 8), North America (*n* = 4), Asia (*n* = 3), and Africa (*n* = 1). All but one [50] listed the number of student participants. The total number of reported students included was 771 (mean 51.4, range 10–205). The year of publication ranged from 2010 to 2022. Most studies (*n* = 13) involved a single interview lasting between 35–90 min. Two studies involved multiple interviews; and one did not report how frequent or long the interviews were. Whether the medical school was graduate entry was rarely reported; however, it could be assumed that North American students were likely to be graduate entry medical students. The setting (medical school year) ranged from 2nd to final year, with most studies involving 4th- or 5th-year students. Only half of the studies reported detailed demographic characteristics. In these studies, between 50–84% of respondents were female. Table 1 lists the characteristics of the included studies.

**Table 1:**
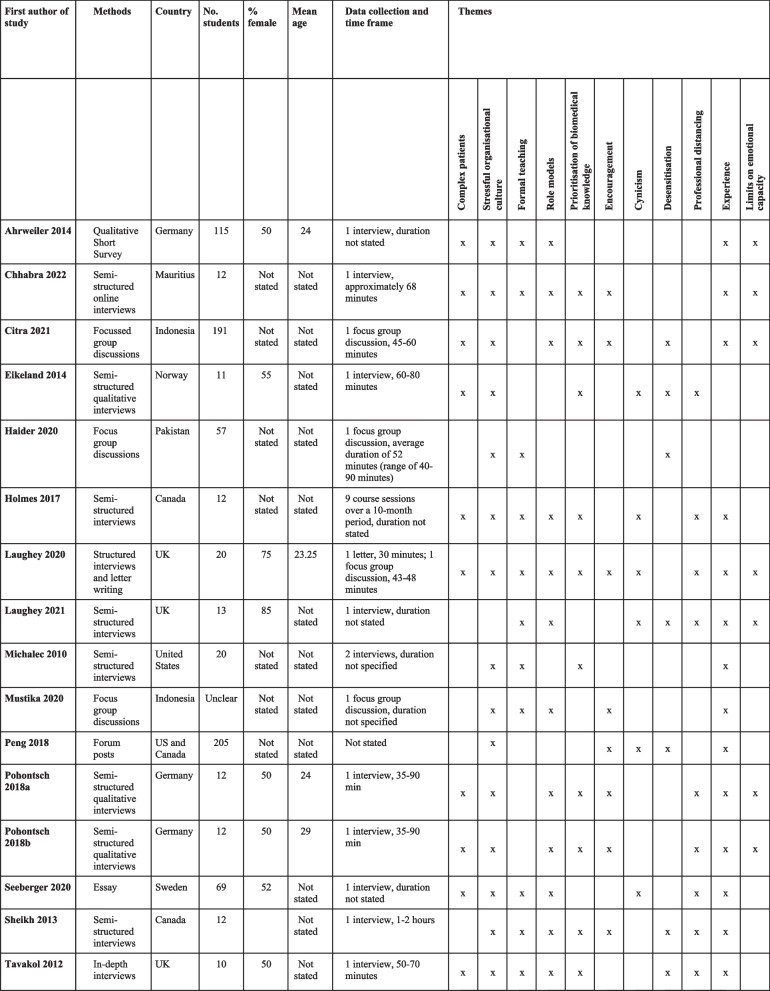
Characteristics of included studies [11–13, 40, 41, 43, 44, 47–51, 53, 55, 57]

### Risk of bias in the included studies

Table 2 includes a summary of the risk of bias assessments. In five of the included studies, it was unclear whether they had obtained ethical approval [11–13, 43, 50]. One study had no areas of concern [48], and the rest had between one (*n* = 3) [12, 47, 55] and 8 (*n* = 1) [43] bias concerns. The most consistent concern (*n* = 14) was whether participants’ voices were adequately represented.

**Table 2:** Risk of bias of included studies

Study	Is there congruity between the stated philosophical perspective and the research methodology?	Is there congruity between the research methodology and the research question or objectives?	Is there congruity between the research methodology and the methods used to collect data?	Is there congruity between the research methodology and the representation and analysis of data?	Is there congruity between the research methodology and the interpretation of results?	Is there a statement locating the researcher culturally or theoretically?	Is the influence of the researcher on the research, and vice- versa, addressed?	Are participants, and their voices, adequately represented?	Is the research ethical according to current criteria or, for recent studies, and is there evidence of ethical approval by an appropriate body?	Do the conclusions drawn in the research report flow from the analysis, or interpretation, of the data?
**Ahrweiler 2014 **[13]	~	+	~	+	+	+	—	~	~	+
**Chhabra 2022 **[40]	+	+	+	+	+	~	—	~	+	+
**Citra 2021 **[41]	~	+	~	+	+	—	—	~	+	+
**Eikeland 2014 **[12]	+	+	+	+	+	+	+	+	~	+
**Haider 2020 **[43]	~	~	~	~	—	+	+	~	~	~
**Holmes 2017 **[44]	+	+	+	+	+	~	~	~	+	+
**Laughey 2020 **[47]	+	+	+	+	+	+	+	~	+	+
**Laughey 2021 **[48]	+	+	+	+	+	+	+	+	+	+
**Michalec 2009** [49]	~	+	+	+	+	+	+	~	+	+
**Mustika 2020 **[50]	~	+	+	+	+	—	—	~	~	+
**Peng 2018 **[51]	~	+	+	+	+	—	—	~	+	+
**Pohontsch 2018a **[11]	+	+	+	+	+	+	~	~	~	+
**Pohontsch 2018b **[11]	+	+	+	+	+	+	~	+	~	+
**Seeberger 2020 **[57]	~	+	+	+	+	—	—	~	+	+
**Sheikh 2013 **[53]	~	+	+	+	+	+	—	~	+	+
**Tavakol 2012 **[55]	+	+	+	+	+	+	+	~	+	+

“ + ” = Yes;”—" = No; “ ~ ” = Unclear

### Results of the thematic syntheses

**Fig. 2:**
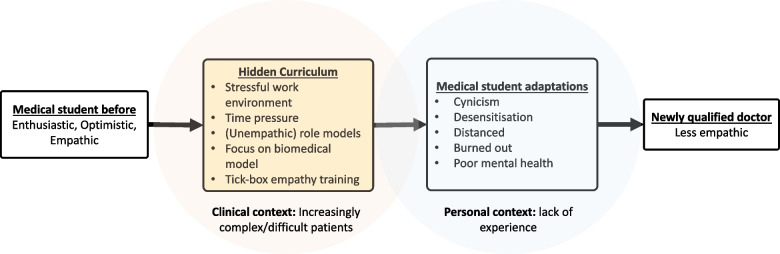
Why empathy declines throughout medical school

Changes in medical student empathy were reported to be influenced (usually reduced) by several factors. Four interpretive themes were developed as part of our synthesis: ‘complexity’, ‘hidden curriculum’, ‘acquired adaptations’, and ‘capacity limits’. Each was derived from 11 (descriptive) sub-themes (see Table 3 for a summary). ‘Complexity’ was derived from the difficulty of relating to the patients’ complexity, especially their socioeconomic situations and diseases. ‘Hidden curriculum’ was derived from ‘stressful organisational culture’, ‘formal teaching’, ‘role models’, ‘prioritisation of biomedical knowledge’, and (lack of) ‘encouragement’. ‘Acquired adaptations’ was derived from ‘cynicism’, ‘desensitisation’, and ‘distancing’. ‘Capacity limits’ was derived from ‘lack of experience’ and ‘limits on emotional capacity’. The themes appeared to be linked, with the hidden curriculum leading to adaptations such as cynicism that decrease empathy (see Fig. 2).

**Table 3:** Themes

Analytical theme	Descriptive theme	Number of studies
**Complexity**	Complex patients	*n* = 10 [11–13, 40, 41, 44, 47, 55, 57]
**Hidden curriculum**	Stressful organisational culture	*n* = 15 [11–13, 40, 41, 43, 44, 47, 49–51, 53, 55, 57]
	Formal teaching	*n* = 11 [13, 40, 43, 44, 47–50, 53, 55, 57]
	Role models	*n* = 12 [11, 13, 40, 41, 44, 47, 48, 50, 53, 55, 57]
	Prioritisation of biomedical knowledge	*n* = 10 [11, 12, 40, 41, 44, 47, 49, 53, 55]
	Encouragement	*n* = 8 [11, 40, 41, 47, 50, 51, 53]
**Acquired adaptations**	Cynicism	*n* = 6 [12, 44, 47, 48, 51, 57]
	Desensitisation	*n* = 7 [12, 41, 43, 44, 48, 53, 55]
	Professional distancing	*n* = 9 [11, 12, 44, 47, 48, 53, 55, 57]
**Capacity limits**	Experience	*n* = 14 [11, 13, 40, 41, 44, 47–51, 53, 55, 57]
	Limits on emotional capacity	*n* = 7 [11, 13, 40, 41, 47, 48]

#### Complexity

As they transition through medical school training, students are exposed to increased patient and social complexity, which was reported to be a barrier to empathy in 10 studies [11–13, 40, 41, 44, 47, 55, 57]. While complexity was based on a single descriptive theme, we noted that it had many intertwined dimensions, ranging from the complexity of patients and diseases to the complexity of socioeconomic circumstances and values.

‘I sometimes find it difficult [to have empathy with] patients who suffer from dementia, are bedridden, need a lot of care or who no longer communicate with the people around them. I also find that one should also consider how much these people might actually still understand of what one communicates.’ [11]

‘Empathy can also make me feel guilty when I struggle to empathise with certain patients such as those with very different views to my own or patients… I feel are difficult.’ [47]

However, exposure to complexity was found to increase empathy in some students.

‘Meeting many patients with different diseases, different backgrounds and different individual needs, preferences and values was considered an important promoter of professional empathy. As a female student (no. 3) wrote: Meetings with patients both increased her ability to assume the patient´s perspective and developed her ability to express her empathy in a way that was adjusted to the individual patient.’ [57]

#### Hidden curriculum

The hidden curriculum (or informal curriculum) has been defined as the subtle, non-formal influence students witnessing (sometimes) unempathic role models, time pressure, the prioritisation of biomedical knowledge and cynicism (as a coping strategy) [59]. The hidden curriculum was explicitly mentioned in eight studies [11, 12, 44, 47–49, 51] and cited by an additional one [40] as a cause of empathy decline.

##### Stressful organisational culture

The working environment in which the students are immersed was noted as being stressful and, in some cases, different from the environment in the earlier phases of medical school. This theme came across consistently within 15 studies [11–13, 40, 41, 43, 44, 47, 49–51, 53, 55, 57]. This culture was caused by specific factors, such as workload, and an overly competitive environment.

‘Professional training was described as being cut-throat and competitive, hindered by administrative policies, long hours and the need to constantly maintain high level performance.’ [51]

##### Formal teaching

In 11 studies, formal empathy teaching was found to impact students’ ability to empathise [13, 40, 43, 44, 47–50, 53, 55, 57]. In most cases, the influence of teaching was believed to have a positive effect.

‘…it is good that they do it [empathy training] in the first and second year because we’re new and we’ve not had that sort of training before. Maybe like just before we start clinical [training]. So if we just had 1 day just sort of recapping things and I think that would be quite useful.’ [55]

However, the impact of the formulaic or ‘tick-box’ approach to empathy that is currently being encouraged in some medical schools to pass exams was found to detrimentally affect students’ ability to empathise.

‘Assessments were deemed to lead to a reductionist, or “tick box” approach to empathy. …in an OSCE [as Objective Structured Clinical Examinations], you’re just trying to tick a box, aren’t you? And you drop in a statement “oh that must be really hard?” and I think there is probably quite a lot of that. But then… everyone is under a lot of stress.’ [48]

##### Role models

In 12 studies [11, 13, 40, 41, 44, 47, 48, 50, 53, 55, 57], the way senior clinicians demonstrated empathy towards patients was reported to influence how students empathised with their patients.

‘Students felt that their empathy had improved only because of the positive role modelling by senior teachers. They mentioned that they learned empathy by observing them. One of the students commented: “I have no doctors in the family, so I came with zero knowledge, and whatever I came to know was because of our teachers and what I observed in the hospital through whatever they taught us. So, we only learn from them.”’ [40]

While positive role models encouraged empathy, negative role models had the opposite effect.

‘…there might be a barrier in so much as they don’t want to have anything to do with their patients, they’d just rather treat them and get on with it, which I don’t think is conducive to the best patient care that you can give someone, but a lot of doctors personally just don’t have great empathy skills or don’t have great communications skills in order to communicate their empathy.’ [55]

##### Prioritisation of biomedical knowledge

While there was no suggestion that biomedical knowledge was unnecessary, the *prioritisation* of theoretical knowledge over soft skills made it more difficult for some students to learn empathy. This was illustrated in 10 studies [11, 12, 40, 41, 44, 47, 49, 53, 55].

‘When one only focusses on the scientific aspect, learning facts by heart aspect, instead of focusing on the human aspect and the person behind the patient, always treating everything as just another case, then one works according to standard procedures. It definitely hinders empathy.’ [11]

##### (Lack of) Encouragement

In eight studies [11, 40, 41, 47, 50, 51, 53], students reported encouragement improved their ability to express empathy. This encouragement could come from peers or seniors.

‘However, most of the students felt motivated when encouraged by senior faculty members.’ [40]

‘FGDs [focus group discussions] among the medical student and resident groups revealed that their peers were the most influential factor in developing their humanistic characteristics. Having peers that reminded them to treat patients as human beings helped them to become humanistic physicians, as the desired behaviours would eventually become habits.’ [50]

However, not all encouragement was positive.

‘[We’re] not appreciated… We give advice… But why they do not listen, nah. So..you ignore yourself, why do we care for you?!’ [41]

#### Acquired adaptations

Students reported developing various ways of coping with the stress of medical school, which in turn influenced their ability to empathise with patients. These were: cynicism, desensitisation, and professional distancing.

##### Cynicism

Six studies reported that students found it challenging to maintain empathy for all patients, so they developed a sense of cynicism to protect themselves from burnout [12, 44, 47, 48, 51, 57].

‘Also, you can become more cynical as well… if you are in GP and the GP is like “this patient coming in just really doesn't help themselves” then that impacts your empathy the other way.’ [47]

##### Desensitisation

In seven studies [12, 41, 43, 44, 48, 53, 55], students’ extended exposure to emotionally taxing experiences throughout their training caused them to become desensitised and consequently reduced their ability to empathise.

‘Since the beginning of the year, we get to see sick patients and get desensitised. We are dealing with very serious patients all the time and we don't have the time to empathise with each patient.’ [43]

##### Professional distancing

Nine studies [11, 12, 44, 47, 48, 53, 55, 57] found that to be able to act professionally, students emotionally distanced themselves from patients, which hindered their ability to empathise with them.

‘Developing a certain emotional distance from the patient, and avoiding too much empathy was widely understood as being a key component of being a professional. One student was very conscious that she should not be a friend, or behave as a family member, but instead create a professional distance. The student vigorously tried to create distance, and avoided thoughts like “What if it was me, or my sister.”’ [12]

#### Capacity limits

A range of factors limited students’ ability to empathise. There was no suggestion that these limits were inherent or insurmountable; however, a lack of experience and emotional capacity limits were reported as barriers to empathy in many of included studies.

##### Experience

Students had a range of previous experiences that affected their ability to empathise when they entered the clinical phase of their education. This theme was apparent in 14 studies [11, 13, 40, 41, 44, 47–51, 53, 55, 57]. The differing experiences included professional experience (exposure to real patients) and personal experience (medical students are generally healthier and from more privileged socio-economic backgrounds than many of the complex patients they treat). The lack of experience can lead to insecurity. Furthermore, the increased pressure of medical school limited opportunities for experiences outside medical school. This, in turn, reduced students’ experiences interacting with patients.

‘I also remember that it is difficult to be empathetic when you do not understand the situation or the disease. I was at the rheumatology ward early in the early part of the course in clinical medicine and had to perform a bedside investigation of a girl of my age who had just received a diagnosis of SLE. She had only had one or two non-severe attacks of the disease, but she had many questions about long-term prognosis and fertility, and she started to cry in front of me. At this time, I did not know much about SLE, medication and the long-term effects of the disease and had never had concrete thoughts about pregnancy or about chronic disease in young people. I found it very difficult to understand her immediately and to act empathetically.’ [57]

However, experience was found to promote empathy in some cases.*‘.. one that can increase empathy is… personal experience, because I had fractured, and I was treated and how it felt, so, me and my fractured patients are tended to care.*’[41]

##### Limits on emotional capacity

In seven studies [11, 13, 40, 41, 47, 48], students’ empathetic abilities were limited due to personal stresses that reduced their emotional availability.

‘There is (..) a limit regarding a person ‘s emotional capacity, [empathy is easier when one] doesn’t have additional personal stress, three friends with problems and a sick father or something else.’ [11]

### Exploring heterogeneity

There was not enough data, and reporting was not sufficiently complete to formally investigate sources of heterogeneity. However, we did note a few potential causes of heterogeneity. Medical students from the US or Canada, who were more likely to be older graduate entry medical students, did not report that life experience and personal maturation impacted their ability to empathise.

While we did not have enough studies from different continents to conduct subgroup analyses by country or continent, the three studies from Asia were represented in all four analytical themes, while the study from Africa was represented in three of the four analytical themes.

### Certainty of evidence

The results of our assessments regarding the overall quality of evidence are presented in Supplementary Table 1. We had a high level of confidence in the evidence for ‘complexity’, ‘stressful organisational culture’, ‘role models’, and ‘prioritisation of biomedical knowledge’. ‘Formal teaching’, ‘encouragement’, ‘desensitisation’, ‘professional distancing’, ‘experience’, and ‘limits to emotional capacity’ were supported by moderate-quality evidence. ‘Cynicism’ was the only theme supported by low-quality evidence, and no themes were supported by very low-quality evidence.

## Discussion

### General interpretation of results

Evidence from 16 qualitative studies revealed a number of themes that explain why empathy appears to change (usually: decline) throughout medical school. The themes identified were remarkably consistent, with all but three supported by at least 50% of the included studies. There was also consistency in the direction of empathy change reported, with most themes related to reasons for a decrease in empathy. The hidden curriculum, particularly a stressful working environment and (sometimes) poor role modelling, led to cynicism, distancing, and desensitisation. This happens in a context where the patients that students are exposed to are increasingly complex (see Fig. 2). Without adequate support and intervention and limited experience and emotional bandwidth, students were unable to overcome the hurdles that stood in the way of maintaining empathy. Many of the themes were linked. Role models seemed to impart the ‘understanding’ that professional distancing is a good thing, and they also sometimes failed to encourage the students. Meanwhile, high workloads (part of the hidden curriculum) contributed to stress and empathy-reducing adaptations, such as cynicism and desensitisation.

Four themes were related to both positive and negative changes in empathy. While inadequate (‘tick box’) teaching and assessment were found to diminish empathy, formal empathy training was found to enhance empathy. Furthermore, while a lack of experience was reported to limit a student’s ability to empathise, if they or their family members had life experiences similar to those of the complex patients they saw, they found it easier to empathise. Similarly, while empathic role models were reported to enhance empathy, unempathetic role models were found to inhibit it. Finally, while exposure to complexity posed a barrier to many students, it provided others with the opportunity to expand their depth of understanding. Encouragement from senior clinicians or peers was found to enhance or inhibit empathy, depending on whether the encouragement was positive or negative.

### Relationship to other evidence

This study is the first synthesis of the reasons why empathy appears to decline in students throughout medical school. Two previous quantitative systematic reviews found that empathy is likely to decline throughout medical school [6, 7]. The authors of one review hypothesised that the reasons for this decline included stress, high workloads, cynicism, and the hidden curriculum [7]. Our results provide firmer evidence that explains this phenomenon and supports the previous authors’ judgments as to why empathy seems to decline throughout medical school.

One study aimed to pinpoint the medical school year where medical student empathy may decline and reported that it happens in the 3rd year [10]. Many of the themes we identified, including a lack of experience and inadequate role modelling, may be more relevant in the clinical years of medicine. This, in turn, suggests that the decline could occur during the clinical phase (which typically starts in year three) of medical school.

Our findings dovetail with a recent study which found that, when comparing standard medical school students with doctor of osteopathy (DO) students in the US [60], empathy among the DO students did not decline to the same degree as in standard medical school students. The authors hypothesised that the reasons for the gentler decline in empathy among DO students involved the features of DO education, such as an emphasis on holistic, patient-centred collaborative care and compassion [60]. Our review corroborates and adds to these findings by demonstrating that an emphasis on the biomedical model seems to dampen empathy.

Our research also mirrors related research, which found that barriers to compassionate care seem to increase as students progress through medical school [61]. Our finding that time pressure and organisational culture contribute to a decline in empathy corroborates the extensive evidence on the inverse relationship between empathy and burnout among healthcare practitioners [2, 3, 62–65] and students [39].

A recent study found that, among graduate entry medical students, those who had undergraduate degrees in humanities or social sciences had higher empathy scores than those with science, technology, engineering, or mathematics degrees [66]. While this association could be explained by a common cause (i.e., those who choose to study humanities could be more empathic), our study provides potential evidence for a causal nature of this association. As a focus on the biomedical model appears to result in empathy decline, studying humanities could protect against this decline.

### Strengths and limitations of the evidence included in the review

The strengths of this review include its novelty, the fact that it followed a published protocol and was reported according to PRISMA, the range of databases searched, and the thematic synthesis. In addition, our qualitative findings addressed the problems associated with quantifying changes in empathy [8, 67]. Our review also has several limitations. Following the Cochrane methodology [68], our search for qualitative studies was narrow. While there is no pragmatic solution to this problem, some studies might have been missed. However, our descriptive and analytic themes were consistent across many of the included studies, which mitigates the impact of this potential limitation. Similarly, the concept of empathy is both poorly defined and overlaps with other related terms, such as compassion and communication. Our strategy for overcoming this problem was to focus on studies that explicitly used the term ‘empathy’. “It is inevitable that some studies that would have been found to concern empathy, may not have mentioned the term explicitly. However, the consistency of the themes identified also mitigates the impact of this potential limitation. The consistency of results notwithstanding, the limited number of studies from outside Europe and North America limits the global generalisability of our findings.

Our conclusions were also limited by the quality and reporting adequacy of the included studies. For example, of the studies that reported demographic characteristics, between 50–84% of respondents were female. As female practitioners seem to be better at empathizing with patients than their male counterparts [5], this could have biased our results (in an unknown direction). Poor reporting of demographic characteristics renders any speculation about the influence of this factor impossible to specify with any precision. Furthermore, the data were not rich enough to formally explore whether there were differences between graduate and undergraduate medical schools, between medical schools that separated the curriculum into pre-clinical and clinical phases, or those that introduced clinical exposure earlier on. It could be that students who attend medical schools that emphasise early patient contact experience less of a decline in empathy. Future research could investigate this further. We hypothesise that students who attend schools with more traditional pre-clinical/clinical structures have a stronger focus on the underpinning biomedical science, resulting in an accentuated focus on biomedical knowledge, which we found to be a cause of empathy decline. Finally, there is debate regarding the use of measures of intercoder reliability for qualitative research [69], which we did not use.

### Implications of the results for practice, policy, and future research

Researchers can use our findings to develop educational interventions that reduce, stop, or even reverse the decline in medical student empathy. These interventions should be developed and evaluated using evidence-based methodology [14, 18] and explore potential cultural and ethnic differences in the expression and interpretation of empathic care [70]. National and international bodies that govern medical school curricula, such as the GMC in the UK and the Liaison Committee on Medical Education in the US and Canada, can use this information to prioritise the modification of medical school curricula to prevent or reverse empathy decline.

### Conclusions

Medical students provided a variety of reasons regarding why their empathy declines throughout medical school. The root cause of the decline in empathy appears to be a ‘hidden curriculum’, which includes stressful workloads, a lack of peer support, and prioritisation of biomedical knowledge; this leads to cynicism, distancing, and ultimately less empathy. The benefits of empathic care to patients and practitioners suggest that medical schools should prioritize developing an ‘empathic hidden curriculum’ that enhances rather than reduces medical student empathy.

## Supplementary Information

**Additional file 1.** 

**Additional file 2.**

## Data Availability

Data related to this publication has been submitted as supplementary materials. Please contact the corresponding author if additional information is required.
