# Supplementary material for: Why might medical student empathy change throughout medical school? a systematic review and thematic synthesis of qualitative studies
Source: BMC Med Educ. 2023 Apr 24;23:270. doi: 10.1186/s12909-023-04165-9 (PMC10124056; doi:10.1186/s12909-023-04165-9)
Supplement: Supplementary file 1 — Additional file 1. [file 12909_2023_4165_MOESM1_ESM.docx]

**Supplementary File 1. Sample search strategy (OVID Medline)**

1. Empathy/ or empath*.mp. 34040
2. Compassion.mp. 8379
3. Students, Medical/ 41045
4. (medic* adj3 student*).mp. 69694
5. 1 or 2 38849
6. 3 or 4 69694
7. 5 and 61829
8. ((("semi-structured" or semistructured or unstructured or informal or "in-depth" or indepth or "face-to-face" or structured or guide) adj2 (interview* or discussion* or questionnaire*)) or (focus group* or qualitative or ethnograph* or fieldwork or "field work" or "key informant")).tw,kw. or interviews as topic/ or focus groups/ or narration/ or qualitative research/ 475014
9. ((mixed or multi*) adj2 method*).ti,ab. 95691
10. multimethod*.ti,ab. 2265
11. 8 or 9 or 10 544371
12. 7 and 11 426
13. limit 7 to "qualitative (maximizes sensitivity)" 1254
14. 12 or 13 1347
