# Supplementary material for: Why might medical student empathy change throughout medical school? a systematic review and thematic synthesis of qualitative studies
Source: BMC Med Educ. 2023 Apr 24;23:270. doi: 10.1186/s12909-023-04165-9 (PMC10124056; doi:10.1186/s12909-023-04165-9)
Supplement: Supplementary file 2 — Additional file 2. [file 12909_2023_4165_MOESM2_ESM.docx]

**Supplementary Table 1. Systematic Review GRADE CERQual Evidence Profile**

| **Review Finding** | **No. Studies Contributing** | **Assessment of Methodological Limitations** | **Assessment of Coherence** | **Assessment of Relevance** | **Assessment of Adequacy of Data** | **Overall assessment of Confidence in the Evidence** | **Explanation of Overall assessment** |
| --- | --- | --- | --- | --- | --- | --- | --- |
| Complexity | 10 [11-13, 38, 39, 53, 55, 57] [42, 45] | Moderate Concerns: 7 Studies had concerns regarding researcher influence on the study and sampling. 3 Studies had serious concerns regarding researcher influence and impact on the study. 1 Study had no concerns. | Minor Concerns: Complexity is multi-dimensional and non-specific. | Minor Concerns: 6 studies are directly relevant to the review question context.  2 studies had partial relevance.  1 study had a different phenomenon of interest. | No concerns: 2 studies produced superficial level data. 7 studies produced detailed and rich data and there is sufficient data quantity. | High Confidence | No concerns in data adequacy. Minor concerns with relevance of data.  Minor concerns with coherence due to complexity of the reason why patient characteristics cause decline in empathy. Moderate concerns with methodology due to lack of data on researcher affects. |
| Stressful organisational culture | 15 [11-13, 38, 39, 41, 42, 45, 47-49, 51, 53, 55, 57] | Moderate Concerns: 6 Studies had concerns in acknowledging the impact of the researcher on their Study. 5 studies had no or minor concerns relating to either researchers’ role on the study or the congruity between philosophical perspective and methodology. 1 study did not address researcher role due to the structure of the study. | No Concerns: Large breadth of support for this review finding from the underlying primary data. However, the review finding could have been elaborated on slightly to reflect the complex factors within this organizational environment the affect empathy. | Minor Concerns: 4 studies had partial relevance to the context of the review question.  1 study had a different phenomenon of interest.  1 study was of unclear relevance.  7 studies were directly relevant to the context of the review question. | No Concerns: Sufficient detailed rich data and good quantity of data to support this review finding. | High Confidence | No concerns with adequacy and coherence of data.  Moderate concerns with the methodology of the included studies. Primarily due to researcher effects.  Minor concerns with the relevance of the data. |
| Formal teaching | 11 [13, 38, 41, 42, 45-48, 51, 53, 55] | Moderate Concerns: 1 study has concerns regarding ethical approval and there is lack of congruity between their methodology and interpretation of the results. 4 studies did not acknowledge researcher effects on the study results. 4 studies had no or minor concerns regarding acknowledging the impact of the researcher on their study. | Moderate Concerns: Large amounts of data in the primary studies linked the review finding with the data however, the  Review finding does not accurately depict the varying ways that teaching can be seen not only as a benefit but as an inhibitor for empathy.  Is the teaching defined as bedside teaching or standardized patient teaching and what are we defining as teaching? | Minor concerns: 2 studies with partial relevance.  1 study with unclear relevance.  8 studies with direct relevance. | No concerns: Good quantity of data to support and good data richness. | Moderate confidence | Moderate concerns with coherence and methodology of included studies.  4 studies did not acknowledge researcher effects on the study results which is a limitation for the methodology.  Coherence has limitations in the complexity of factors that support this review finding not being represented by the review finding itself. |
| Role models | 12 [11, 13, 38, 39, 46, 48, 51, 53, 55, 57]  [42, 45] | Moderate Concerns: 6 studies did not address the role of the researcher and its impact on the study. 2 studies had minor limitations regarding addressing researcher effects. 2 studies had no or minor limitations in design with only slightly unclear nature of researcher role. | No concerns: Very strong link between the underlying data and the review finding. This finding could be improved slihglty by changing it to positive role models enhance empathy versus negative role modelling forming a barrier against empathy. | Minor concerns: 1 study with unclear relevance.  1 study with partial relevance.  9 studies with direct relevance to the context of the review question. | No Concerns: 11 studies contributed to this review finding.  Detailed rich data from included studies as well as a good quantity of data available. | High confidence | Moderate concerns in methodology.  No concerns with adequacy and coherence.  Minor concerns with relevance. |
| Prioritisation of biomedical knowledge | 10 [11, 12, 38, 39, 42, 45, 47, 51, 53, 57] | Moderate Concerns: 3 studies had limitations in addressing the researcher’s role.  2 studies had minor limitations in assessing the researchers influence on the study. 1 study had a very small sample size so may not be as representative of our study population as desired. 1 study had no limitations. 1 study had minor limitations regarding congruity between research methodology and intended qualitative approach. | No concerns: This is a review finding that accurately links together the primary underlying data and creates a very strong link. | Minor Concerns: 8 studies with direct relevance to the review question context.  1 study with unclear relevance to the review question context. | No Concerns: 9 Studies contribute to this review finding.  Detailed rich data to support this review finding and a good quantity of studies to support this review finding. | High confidence | Moderate concerns with methodological limitations.  No concerns with coherence and adequacy of data.  Minor concerns with relevance. |
| Encouragement | 8 [11, 38, 39, 45, 48, 49, 51, 57] | Moderate Concerns: 1 study had no concerns.  1 study had minor limitations in not acknowledging the impact of the researcher on the studies. 5 studies had moderate issues with identifying the impact of the researchers’ background and role on the study. | Moderate Concerns: There was variable support among the included studies for this review finding.  1 study does not support this review finding at all.  3 studies had incomplete data to support this review finding.  3 studies had clear links between the review finding and its primary data. | Moderate concerns: 4 studies were directly relevant to the context of the review question.  1 study of unclear relevance to the context of the review question.  1 study had a different context from the review question in its assessment of empathy decline.  1 study of partial relevance with different population of interest as it included residents and junior or senior clinical teachers. | No concerns: 7 studies contributed to this review finding.  6 studies had rich and detailed data.  1 study had superficial level thin data.  Overall there is a good level of richness and good number of studies supporting this review finding. | Moderate Confidence | Moderate concerns in methodology and coherence and relevance of the data.  This is due to inability to acknowledge researcher effects on the study.  Issue with coherence is lack of complete data in some included studies to support an association between the data and the review finding.  No concerns with adequacy of data. |
| Cynicism | 6 [11, 41, 44, 45, 48, 54] | Minor Concerns: 2 studies had concerns regarding researcher impact on the study or their theoretical background. 3 studies had no or minor limitations with researcher influence on the study. 1 study had no concern | Serious Concerns: 1 study has a cogent link between the review finding and underlying data.  3 studies had incomplete data to support the relationship between review finding and the underlying data.  1 study has oversimplified the variety of underlying data to support this review finding. | Moderate Concerns: 3 studies with direct relevance.  1 study with unclear relevance.  1 study with partial relevance. | Minor Concerns: Non-sufficient data as 1 study did not contain data on cynicism.  Data richness was affected by the study design of 2 studies. | Low confidence | Serious concerns with coherence.  Incomplete data of the impact of cynicism in 3 studies.  Also, oversimplification of the various factors associated with cynicism as coping strategy. |
| Desensitisation | 7 [39, 41, 42, 45, 46, 51, 53] | Moderate Concerns: 1 study had concerns regarding their congruity between methods and intended objectives. 1 study had concerns with the impact of the researcher on their study.  2 studies had minor limitations regarding participant representation.  1 study had no concerns. 2 study had minor concerns with researcher impact on the study. | Moderate Concerns: 3 studies had a clear link between review finding and phenomenon of interest.  4 studies had oversimplified the variety of factors that are associated with exposure.  1 study has no or incomplete data in terms of relationship. | Minor Concerns: 5 studies with direct relevance in context of review question.  QS14 with partial relevance.  1 study with unclear relevance. | No Concerns: 7 studies contributed to this review finding.  Sufficient data richness and sufficient data quantity. | Moderate confidence | Minor concerns with relevance and no concerns with adequacy of data.  Moderate concerns with coherence due to oversimplification of the complexity of the factors associated with exposure.  Moderate concerns with methodology due to lack of participant representation and lack of reflection of the researcher influence on the study. |
| Professional distancing | 9 [11, 12, 42, 45, 46, 49, 51, 53, 55] | Moderate Concerns: 2 studies had  concerns with impact of the researcher on their study.  6 studies had no or minor concerns with researcher influence on the study. 1 study did not address researcher role due to the structure of the study. | Minor Concerns: 3 studies had a clear and cogent link between the data and the review finding.  2 studies had oversimplified the variety in the data supporting this review finding.  3 studies had incomplete data to support the review finding. | Minor Concerns:  7 studies with direct relevance in context.  1 study with partial relevance.  1 study with unclear relevance. | Moderate Concerns: 9 studies contributed to this review finding.  Good quantity with limited richness of data to support this review finding. | Moderate Confidence | Minor concerns with coherence and relevance of data.  Moderate concerns with methodology due to 1 study that did not address the researcher’s role due to the structure of the study.  Moderate concerns with adequacy due to thin data supporting this review finding. |
| Experience | 14 [11, 13, 38, 39, 42, 45-49, 51, 53, 55, 57] | Moderate Concerns: 1 study had minor limitations in influence of the researcher.  6 studies with no acknowledgment of the researchers influence or background on the study.  All studies had minor limitations in participants and whether they were adequately represented. | Minor concerns: 4 of the included studies had incomplete data to support a relationship between the review finding and the phenomenon of interest.  Most of the included studies had large amounts of data that clearly links between the review finding and the phenomenon of interest although there may be slight oversimplification of how exactly experience can impact on empathy. | Minor  Concerns: 6 studies are directly relevant to the context of the review question.  1 study had a mixed methods approach.  1 study was of unclear relevance.  1 study utilised critical reflective essays as aside to interview.  2 studies included tutors as well so different population of interest.  1 study had a different phenomenon of interest. | No concerns: 13 studies contributed to this review finding.  3 studies with superficial level thin data.  10 studies with detailed and rich data.  Adequate data richness and data quantity. | Moderate  Confidence | Moderate concerns in methodology due to limitations in adequate participant representation.  There is also limitations in acknowledging the researchers role and influence on the study.  No concerns with data adequacy.  Minor concerns in relevance due to variations in population of interest and methods of data sampling employed by a few of the included studies. |
| Limits to emotional capacity | 7 [11, 13, 38, 39, 45, 46, 57] | Moderate Concerns: 1 study had no r limitations.  3 studies had minor limitations in addressing the participants and their voices. 3 studies had moderate concerns in the influence of the researcher and their background on the outcomes of the study. | Minor concerns:  One study has ambiguous data to support a link between the review finding and the phenomenon of interest.  Majority of other included studies had a clear link between emotional capacity as an influence on empathy and the phenomenon of interest. | Minor Concerns: 4 studies had direct relevance to the context of the review question.  1 study included clinical tutors so had a different population of interest.  1 study had a different context regarding how they assessed empathy decline using letters. | No concerns: 6 studies contributed to this review finding.  5 studies provided detailed and rich data.  1 study employed a questionnaire survey strategy which did not yield detailed or rich information.  Adequate data richness and data quantity observed. | Moderate Confidence | No concerns with data adequacy.  Minor concerns in coherence due to ambiguity in relationship between included data and review finding in one study.  Moderate concerns in methodology due to limitations in addressing the participants and limitations in acknowledging the researchers background and role on the outcomes of the study.  Minor concerns with relevance due to 1 included study including clinical tutors so had a slightly different population of interest that they were studying. |
